# Identification of Sarcopenia in Amazonian Elderly

**DOI:** 10.1093/geroni/igaa057.043

**Published:** 2020-12-16

**Authors:** Myrian Faber

**Affiliations:** University of the State of Amazonas, MANAUS, Amazonas, Brazil

## Abstract

This research has applied the SARCF to identify the presence of sarcopenia in 236 women aged 66.27 ± 5.76 year from Amazonas, who have attended activities aimed at the elderly. The SARCF analyzes five objective variables for assessing sarcopenia, related to the functional pattern (strength, walking, sitting and getting up from a chair, climbing stairs and falls). Functional patterns are scored to independence = 0, some dependence = 1 and a lot of difficulty or functional disability = 2. Regarding falls during the year; none = 0, ≥ 1 ≤ 3 = 1 and ≥ 4 = 2. Values ≥0≤4, suggest absence of sarcopenia, ≥4≤10 sarcopenia. Results has revealed 100 (42.37%) elderly women without difficulties to carry objects weighing 5 kilos, 130 (55.31%) have presented some difficulty and 6 (2.55%) couldn’t do it so. As for walking, 108 (45.76%) haven’t needed help, 128 (54.23%) have needed some help. Regarding to sit and get up from a chair, 17 (7.20%) haven’t needed help, 218 (92.37%) have needed help and 1 (0.42%) couldn’t do it. Climb stairs 17 (7.20%) haven’t needed help, 173 (73.30%) have needed help and 46 (19.49%) couldn’t do it so. Concerning about falls, 150 (63.55%) elderly women haven’t fallen, 57 (24.15%) have fallen between one and three times and 29 (12.29%) have fallen more times. The final SARCF score has revealed a sarcopenia prevalence in 54% of the surveied population; CI = 0.30, CORREL = 0.90 (54.05%) sarcopenic; 6 (2.55%) pre-sarcopenia, 24 (18.9%) sarcopenia, 24 (18.9%) severe sarcopenia.

